# Quality Improvement Coaching for Human Papillomavirus Vaccination Coverage: A Process Evaluation in 3 States, 2018–2019

**DOI:** 10.5888/pcd17.190410

**Published:** 2020-10-08

**Authors:** Jennifer Leeman, Victoria Petermann, Jennifer Heisler-MacKinnon, Adam Bjork, Noel T. Brewer, Brigid K. Grabert, Melissa B. Gilkey

**Affiliations:** 1 School of Nursing, University of North Carolina, Chapel Hill, North Carolina; 2 Lineberger Comprehensive Cancer Center, University of North Carolina, Chapel Hill, North Carolina; 3 Department of Health Behavior, Gillings School of Global Public Health, University of North Carolina, Chapel Hill, North Carolina; 4 Immunization Services Division, Centers for Disease Control and Prevention, Atlanta, Georgia; 5 United States Public Health Service, Commissioned Corps, Rockville, Maryland

## Abstract

**Purpose and Objectives:**

Quality improvement (QI) coaching improves human papillomavirus (HPV) vaccination coverage, but effects of coaching have been small, and little is known about how and when QI coaching works. To assess implementation outcomes and explore factors that might explain variation in outcomes, we conducted a process evaluation of a QI coaching intervention for HPV vaccination.

**Intervention Approach:**

QI coaches received tools and training to support 4 core coaching competencies: 1) expertise in using clinic-level adolescent vaccination data to drive change, 2) knowledge of the evidence base to support change in HPV vaccination practice, 3) familiarity with improvement strategies and action planning, and 4) skill in building relationships.

**Evaluation Methods:**

Our mixed methods evaluation involved collecting quantitative data through effort-tracking logs and gathering qualitative data through in-depth interviews with QI coaches (N = 11) who worked with 89 clinics in 3 US states. Data were collected on implementation outcomes and on contextual factors that might explain variations in those outcomes. Implementation outcomes included adoption by clinics, reach to providers and staff (ie, participation in the coaching visit), and implementation fidelity.

**Results:**

States achieved either high adoption or high reach, but not both. For example, state A had high adoption with 94% of clinics accepting a coaching visit, but low reach with a median of 1 participant per clinic. In contrast, state C had lower adoption (29%, *P* < .01) than state A but higher reach (median of 4 participants per clinic, *P* < .01). Generally, states had high coaching protocol fidelity with the exception of advising on strategies and action planning. QI coaches described factors that might explain these variations, including strength of relationships with clinic staff and whether they recruited clinics directly or through large clinic networks.

**Implications for Public Health:**

Our findings have implications for the design of future QI coaching initiatives, including how coaches recruit clinics to ensure full clinic engagement, refinements to coaching visits, and how QI coaches can effectively engage with clinic networks. Findings could inform future QI coaching interventions to strengthen their impact on public health.

**Summary**

**What is already known on this topic?**

Quality improvement (QI) coaching is effective in improving clinic human papillomavirus (HPV) vaccination coverage; however, improvements are generally small, and little is known about factors influencing QI coaching effectiveness.

**What is added by this report?**

We report implementation outcomes for a QI coaching intervention and qualitative findings on factors that might explain when coaches are successful at 1) persuading clinics to adopt the intervention, 2) reaching clinic staff and providers during a coaching visit, and 3) implementing coaching protocols with fidelity.

**What are the implications for public health practice?**

Results of our evaluation advance understanding of factors that might influence the successful implementation of QI coaching and inform the development of future coaching interventions.

## Introduction

Persistent human papillomavirus (HPV) infection leads to over 34,000 new cancer diagnoses per year in the United States (1). HPV vaccination is highly effective at preventing HPV cancers, yet only 51% of US adolescents aged 13–17 have received the recommended number of doses (2). The Centers for Disease Control and Prevention (CDC) provides funding for immunization quality improvement (QI) coaching to 61 state, local, and territorial immunization programs, with the goal of increasing immunization rates for routinely recommended vaccines, including HPV vaccine (3). CDC provided this coaching through the AFIX (Assessment, Feedback, Incentives, and eXchange) program, which was replaced by IQIP (Immunization Quality Improvement for Providers) in July 2019. The AFIX program engaged staff in state and regional health departments to deliver QI coaching to improve clinics’ immunization practices (2). QI coaching, also referred to as “practice facilitation,” is an evidence-based implementation strategy defined as a process of interactive problem solving and support that occurs in the context of a recognized need for improvement and a supportive interpersonal relationship (4).

Researchers at the University of North Carolina (UNC) developed an intervention that provides tools and training to support the delivery of QI coaching to improve clinics’ HPV vaccination rates (5). In a previous study, we found that the intervention demonstrated a small but significant improvement in HPV vaccination rates in clinics that received HPV vaccination QI coaching, as compared with those that did not (6,7). In the evaluation presented here, we explore factors that influenced the implementation of HPV vaccination QI coaching, with the goal of further refining the intervention.

## Purpose and Objectives

The UNC research team sought to compare the effectiveness of HPV vaccination QI coaching to clinical medical education by conducting a randomized controlled trial in primary care clinics in 3 states (clincaltrials.gov NCT 5108275). The purpose of this process evaluation was to assess the implementation outcomes for the QI coaching aspect of the trial and explore contextual factors that might explain variations in implementation outcomes across the states.

## Intervention Approach

The coaching intervention provided tools and training to support QI coaches who worked in CDC-funded AFIX programs in state or regional departments of public health. AFIX-based QI coaches typically were trained in public health or nursing and made an annual 1–2 hour, in-person coaching visit to a subset of primary care clinics in their geographic regions, following the in-person visit with additional coaching by email or telephone. CDC originally designed the AFIX program to improve vaccination coverage for infants and young children and only later expanded it to improve adolescent vaccination coverage. Given persistently low rates of HPV vaccination, the goal of the HPV QI coaching intervention was to provide coaches with additional training and tools to improve HPV vaccination rates for adolescents.

QI coaching is a widely tested implementation strategy. In a systematic review of 22 studies involving 1,429 clinics, reviewers found that clinics that received QI coaching were more likely to adopt evidence-based guidelines than those that did not (8). The activities involved in QI coaching vary across studies but generally require the 4 core competencies of 1) expertise in using data to drive change, 2) knowledge of the evidence base that drives the change in practice, 3) familiarity with available strategies to implement change, and 4) skills in relationship building (9). UNC’s QI coaching tools for HPV are freely available on the project website (https://www.hpviq.org). The tools are designed to support the 4 core coaching competencies and include an immunization report card template to increase QI coach competence in using data to drive change, a PowerPoint presentation to support clinic education on the evidence base driving the change in practice, a menu of recommended improvement strategies to implement change, and protocols for guiding clinics in action planning to initiate change. 

**Immunization report card template**. The report card template provided QI coaches with a tool that supports their efforts to use data to drive change (Figure). Coaches used the template to translate data from the state’s immunization registry into a report card that provides feedback on a clinic’s current coverage or percentage of adolescents vaccinated for HPV, as compared with 2 other adolescent vaccines — meningococcal conjugate vaccine and the tetanus toxoid, reduced diphtheria toxoid, and acellular pertussis (Tdap) booster. Assessing and providing feedback on performance is an evidence-based implementation strategy. A Cochrane review found that assessment and feedback generally led to modest (4%–7%) clinically important improvements in pediatric health outcomes (10). Coaches also used the report card to prompt clinics to set specific goals for improvement.

**Figure:**
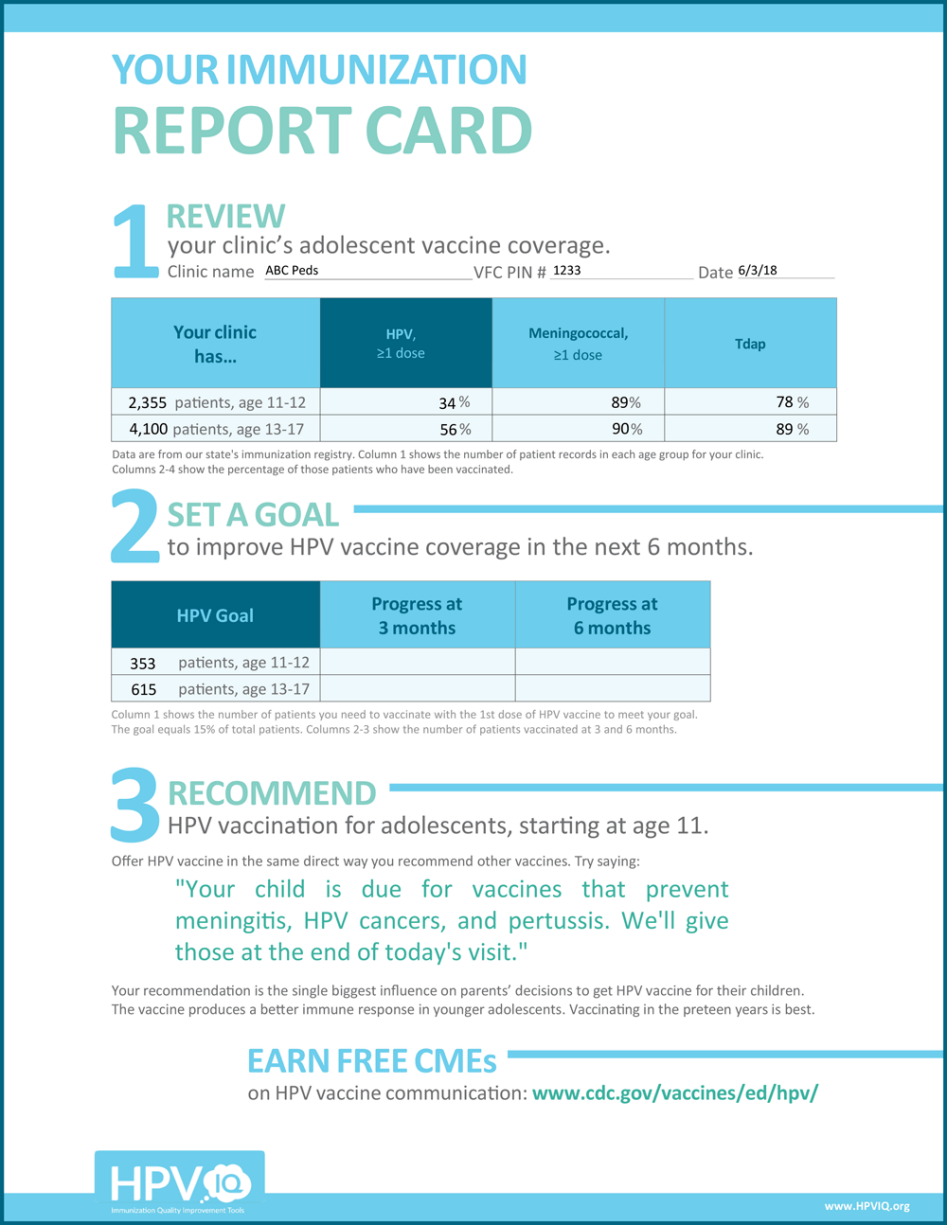
Template for coaches’ immunization report card. Source: **https://www.hpviq.org**.

**Presentation**. Coaches received an HPV PowerPoint slide set to present to clinic providers and staff. The 28-slide presentation began with an overview of the evidence base in support of HPV vaccination as an effective means of preventing cancer in both males and females. The presentation then guided coaches through each step of the QI coaching session, including reviewing the report card, setting a goal for improvement, selecting strategies to improve HPV vaccination, and creating an action plan.

**Menu of recommended improvement strategies.** CDC’s standard AFIX protocols provided QI coaches with a list of 19 improvement strategies that varied in feasibility and potential to increase HPV vaccination rates in clinics. The research team reviewed the list and identified strategies with the greatest potential for feasibility and improvements, based on input from academic and practice-based stakeholders with expertise in HPV vaccination and the AFIX program. As a result, the coaching protocols specified 1 primary strategy (advising providers to recommend same-day HPV vaccination for all patients aged ≥11 years) and 3 secondary strategies (reviewing CDC guidelines with all immunization staff, training front desk staff on scheduling, and establishing standing orders).

**Protocols for guiding clinics in action planning**. The PowerPoint presentation included slides that prompted participants to identify who is responsible for specific vaccination roles in their clinic, such as scheduling appointments, reviewing and flagging charts, and prescribing vaccines. It also prompted participants to start planning how they would work with people identified to improve clinic vaccination coverage.

AFIX encouraged QI coaches to invite additional clinic staff to participate in the 1-time coaching visit. To increase participation and motivate providers to attend, the intervention offered clinical medical education (CME) credits.

To support QI coaches in using the tools and protocols, the research team engaged 8 coaches and 5 of their supervisors in a 2-day, in-person training. The research team then engaged coaches in weekly conference calls to review coaching and data collection protocols and to solve problems that challenged implementation as they were encountered.

## Evaluation Methods

This mixed methods evaluation involved the collection of quantitative data through effort-tracking logs, and qualitative data by using in-depth interviews with QI coaches who worked in AFIX programs in 3 states, from 2018 to 2019. The University of North Carolina Institutional Review Board reviewed the trial and classified it as exempt.

We selected 3 states to participate in the trial on the basis of their geographic diversity, robust state immunization registries, active AFIX programs, and interest of AFIX program staff. These states were in the southwest (state A), the northeast (state B), and the midwest (state C). Baseline HPV vaccine initiation coverage (for patients aged 13–17 y) was 67.2% for state A, 69.8% for state B, and 64.1% for state C (2).

In each state, clinics were eligible for inclusion if they were pediatric or family medicine practices, Vaccines for Children providers, had between 200 and 7,000 patients (aged 11–17 y), and had baseline HPV vaccine initiation coverage below 85%. The federally funded Vaccines for Children program provides free vaccines to high-priority populations, including children who are under- or uninsured. Clinics were ineligible if they belonged to a network with over 30 clinics or were pharmacies or school health clinics. States A and C conducted work statewide, while state B worked in 3 large counties. AFIX visits were open to providers, other members of the primary care team including nurses and medical assistants, and administrative staff.

We collected data on the implementation outcomes of clinic adoption, providers and staff reach, and QI coaching fidelity (11), and on contextual factors that might explain variations in those outcomes. AFIX staff in each state maintained a tracking log of the number and proportion of clinics that agreed to participate (ie, adoption) and the number of staff and providers who participated in each visit (ie, reach). Providers were physicians, nurse practitioners, and physician assistants. All other participants were classified as staff. A researcher conducted in-depth phone interviews with QI coaches in each state to assess how they used the HPV coaching tools (fidelity) and to explore contextual factors that might have contributed to variations in implementation outcomes. Interviews followed a semi-structured interview guide that queried coaches about their experiences recruiting clinics and providers for participation in an HPV QI coaching visit and how they delivered QI coaching. The focus of the guide was the coaches’ use of recommended tools and fidelity to protocols.

For quantitative data, we compared adoption and reach among the 3 states. We determined if adoption rates varied across states by using logistic regressions followed by Wald tests. We determined if reach to providers and staff (number per clinic who attended AFIX visit) varied across states by comparing rank means using the Kruskal–Wallis and Dunn tests because of skewing of data. Because of the skewed nature of those data, we report medians rather than means for reach. For qualitative data, we recorded interviews, with consent of participants, and transcribed them. We used content analysis to analyze data (12). We developed a set of codes for implementation outcomes and contextual factors that influenced outcomes. For implementation outcomes, codes specified performance of core components of HPV QI coaching, including distributing the clinic report card, setting a goal for improvement, sharing the presentation, selecting improvement strategies, and creating an action plan. During the coding process, codes were developed as needed to fully capture all relevant contextual factors. Coders used ATLAS.ti (Scientific Software Development GmbH), a qualitative software management program, to code the interviews. Coders met to compare and reconcile coding. Once coding was complete, data were put into a matrix that organized findings by QI coach and state, and the research team identified themes related to factors influencing implementation outcomes and how those factors varied across states.

## Results

The total sample for our process evaluation (N = 11) consisted of 9 AFIX QI coaches and 2 supervisors who provided HPV QI coaching to 89 clinics in 3 states. State A had 2 QI coaches with a mean of 11.0 years of experience (range, 4–18 years), state B had 5 QI coaches with a mean of 2.3 years of experience (range, 1–6 years), and state C had 2 QI coaches with a mean of 2.3 years of experience (range, 1.5–3 years).

### Implementation Outcomes

**Clinic adoption of HPV QI coaching.** Overall, 63% of invited clinics agreed to participate in QI coaching and completed a coaching visit. Adoption was higher in states A and B, as compared with state C (both *P* < 0.01), with 30 of 32 (94%) clinics adopting in state A, 40 of 44 clinics adopting in state B (91%), and 19 of 65 (29%) adopting in state C.
**Staff and provider reach.** A median of 2 providers and other staff from clinics participated in the QI coaching visit. The total number of participants varied across all 3 states. AFIX visits in state A had a median of 1 participant per visit, and all were staff. State B had a median of 2 participants (1 staff and 2 providers), and state C had a median of 4 participants (4 staff and 1 provider).
**QI coach fidelity to coaching protocols.** QI coaches consistently reported that they used the report card to provide feedback on clinic vaccination data and worked with clinics to set a goal for improving vaccination rates over the next 6 months. Coaches in states B and C reviewed the presentation with participants in the coaching visit. In state A, coaches converted the information into a 1-page handout that they reviewed with participants. Across all 3 states, coaches reported that they worked with clinics to select specific strategies for improving their HPV vaccination coverage. However, coaches did not consistently promote the short list of recommended strategies from the coaching protocol. When interviewed, one-third of QI coaches could not name any of the 4 recommended strategies, and most coaches continued to recommend strategies from the longer list of 19 from the standard AFIX protocol. Only the QI coaches in state C reported conducting action planning with clinics and reported that it was fairly limited. QI coaches in states A and B reported conducting no action planning (Table 1).

**Table 1:** Quality Improvement Coach Fidelity to Human Papillomavirus Vaccination Coverage Improvement Tools and Protocols

Tool	State A	State B	State C
Report card: Distribute a one-page summary of vaccination coverage rates.	Yes	Yes	Yes
Goal setting: Set a 6-month goal for improving vaccination coverage rates.	Yes	Yes	Yes
Slide presentation: Present data on the benefits of vaccination.	Partial	Yes	Yes
Improvement strategy selection: Select strategies to improve vaccination coverage rates.	Partial	Partial	Partial
Action planning: Identify specific next steps that clinic staff will take.	No	No	Partial

### Factors that might explain variations in implementation outcomes

Analysis of the qualitative data suggested several factors that might explain variability in the 3 implementation outcomes (Table 2) of clinic adoption, staff and providers reached, and QI coach fidelity to intervention tools and protocols, as follows.

**Table 2:** Possible Factors in Outcome Variation in QI Coaching to Improve Human Papillomavirus Vaccination Coverage

Factors	Outcomes
Clinic adoption (ie, agreeing to participate in the QI coaching)	Strong relationships between quality improvement coaches and clinic staff were key to gaining entry to the clinics.
	Low turnover rates for both quality improvement coaches and clinic staff contributed to the strength of the relationship.
	Presenting quality improvement coaching as a requirement facilitated adoption.
	Large clinic networks were a barrier to gaining entry to the clinics.
Staff and providers reached (ie, participation in the human papillomavirus coverage coaching visit)	Working with large clinic networks facilitated reach to staff and providers.
	Scheduling visits with medical assistants was a barrier to reaching other staff and providers.
	Summer was a difficult time to reach providers.
	Not all coaches offered clinical medical education (CME) credits and reported mixed perceptions of the effectiveness of CME as an incentive.
QI coach fidelity to coaching tools and protocols	QI coaches who perceived a need to change their current approach reported greater fidelity (ie, tension for change).
	QI coaches who were knowledgeable of QI coaching tools and protocols reported greater fidelity.
	QI coach perceptions of clinic staff and providers participating in the coaching visit (needs and capacity) might have affected fidelity.
	QI coach perceptions of the utility of coaching tools might have explained fidelity.

Abbreviation: QI, quality improvement.

**Clinic adoption.** QI coaches perceived strong relationships with clinic staff as key to gaining entry to the clinics. In state A, where coaches reported strong relationships with clinic staff, clinic-level adoption was 94%. Low turnover rates of both AFIX coaches and clinic staff in state A may have contributed to the strength of the relationships. As one of the coaches in state A reported, “When I call them, they see my name. I've been here for 18 years. A lot of the people know me already, so they know when I call, I'm like, ‘Guess what? It's time for me to come out again,’ and they'll be like, ‘All right.’”
In states A and B, coaches reported that they promoted clinic adoption by presenting the coaching visit as a required meeting, with some coaches adding that the visit is required for participation in the Vaccines for Children program. Coaches in state C did not tell clinics they were required to participate in an HPV vaccination coaching visit, which may have contributed to low adoption rates in that state. The prevalence of large clinic networks in state C also might have contributed to a low adoption rate. More than 85% of the clinics in state C were part of a network, and more than 10% of those networks involved 11 or more clinics. In contrast, in state A, none of the clinics were part of a network, and in state B, 52% of clinics were part of networks, most of which (>94%) were small with 4 or fewer clinics. QI coaches in state C reported that they had difficulty directly recruiting clinics that were part of a network. Instead, they had to navigate the network’s multilevel hierarchy to identify an individual with the authority to approve a visit. As one QI coach from state C noted, “And then there's also a lot of big systems where the person you call at the clinic is not authorized to say, ‘Yes, you can come in and talk to our providers.’ And so, [we] may have to go up the chain, but often gets lost…” In state C, administrative personnel often contacted clinics to schedule the HPV vaccination coaching visit, as compared with the other 2 states where most scheduling was done by the QI coaches.
**Reach to providers and staff.** Although state C had the lowest clinic adoption rates, it had the highest average number of staff and providers participating in coaching visits. In contrast, state A had the highest clinic adoption rates and the lowest rate of staff and provider participation in coaching visits. One factor that might account for this is the staff with whom the coach scheduled the visits. In state C, coaches often scheduled visits with a higher-level representative of the healthcare system, such as a quality improvement leader. In state A, QI coaches scheduled their visits with medical assistants in individual clinics who might have lacked influence in the clinic and been unable to persuade providers to attend the visit.
Across all states, time of year appeared to influence provider participation. QI coaches reported that providers were least available to participate in visits during the summer when vacations reduced the number of providers and staff in the clinic; however, workload increased because of sports- and school-related physical examinations. Only a few of the QI coaches viewed CME as an effective incentive to get providers to participate in the visit. Several QI coaches were either not aware that CMEs were available as an incentive or did not know how to request the CME. Several QI coaches reported that, although physicians typically did not participate, they often came in for at least part of the visit.
**QI coach fidelity to HPV coaching protocols**. Factors that appeared to influence QI coaches’ overall fidelity to the HPV vaccination coaching protocols were perceived tension for change, knowledge of and beliefs about the HPV vaccination coaching protocols, perceptions of the needs and capacity of the person participating in the QI coaching visit, and perceptions of the HPV vaccination coaching tools. Coaches who provided high fidelity coaching perceived a need to change their current approach to QI coaching (ie, tension for change). QI coaches in states B and C acknowledged that their QI coaching could be improved, whereas those in state A were content with their current approach and saw little need to change. QI coaches in state A had the most years of experience providing QI coaching and the greatest success persuading clinics to adopt an AFIX visit, both of which might have contributed to a low perceived tension for change.
Knowledge of HPV vaccination coaching protocols varied among coaches, which appeared to limit their ability to deliver the protocols with fidelity. Knowledge of the protocols did not appear to vary by state, but did vary by whether coaches were among the 8 who attended the 2-day training on HPV coaching. In many interviews, when asked about their process of advising clinics on the selection of improvement strategies, QI coaches who did not attend the training reported that they suggested strategies other than those on the list of strategies from the HPV QI coaching protocol.

QI coaches also described how they tailored delivery to match their perceptions of the needs and capacity of clinic staff and providers. This was particularly salient in state A, where medical assistants were the only participants in AFIX visits. QI coaches reported that their perceptions of participant needs and capacity particularly influenced use of the presentation slides and action planning. For example, QI coaches skipped sections of the slides if they felt that the content was already well known. Similarly, they skipped action planning if they felt the person they were meeting with lacked the capacity to make changes to clinic processes. Low fidelity to protocols for coaches working with clinics to develop an action plan was largely due to the perception that action planning fell outside the role of the person participating in the coaching visit. In the words of a QI coach, “[Clinic staff] weren’t able to see kind of beyond their role and to talk about other people’s roles.” In a few cases, reviewers stated that clinics were already performing well and did not perceive a need to participate in efforts to improve their HPV vaccination rates.

QI coaches’ positive perceptions of the report card, goal setting protocols, and the presentation slides appeared to encourage their relatively high levels of fidelity in the use of those tools. Across all 3 states, QI coaches reported appreciation for the report card’s clarity and conciseness. They all reported using the report card during the coaching visit; however, their perception of the utility of the report card was dampened by clinic staff and provider skepticism about the accuracy of the data reported. Data came from the state immunization registry rather than the clinic’s electronic health records. In some cases, clinic electronic health records lacked a direct interface with the state registry, and delays could occur in clinics uploading their data.

The high level of coaches’ fidelity to goal setting might be explained by their positive perceptions of the way the vaccination report card stated goals as both a number and percentage of patients. As one QI coach noted, “Putting [the goal] into people, rather than a percentage, really helped them understand it. And they’re like, ‘Oh…that’s only like X amount of kids a month.’”

Coaches in all 3 states valued the way the slide presentation outlined the evidence in support of HPV vaccination. In states B and C, QI coaches also valued the PowerPoint format as a way to structure the visit. A QI coach reported, when people “start going off topic, or they start talking about other stuff, it’s really useful to have that PowerPoint to keep the meeting on pace and kind of helping you bring people back.”

## Implications for Public Health

The HPV vaccination coaching intervention provided training and tools to support delivery of evidence-based, data-driven coaching to participating clinics in 3 states. Research has demonstrated that QI coaching is effective in improving the implementation of evidence-based interventions (8); however, the effects are often small and little is known about how, why, and when QI coaching works (13). Implementation outcomes in our study varied across the 3 states, providing an opportunity to explore variations in how QI coaching was delivered and factors that might begin to explain when and why those variations occurred.

Among the 3 states, rates of clinic adoption and provider participation varied such that QI coaches either achieved high adoption or high reach, but not both. In states A and B, where QI coaches gained easy access to clinics, coaches were less successful at getting additional staff to participate in the coaching visit. In contrast, in state C, QI coaches had difficulty gaining entry to clinics but were successful in getting multiple providers and staff to participate. This finding suggests that successful implementation of HPV coaching depends not only on clinic adoption of the approach but also on clinic readiness to improve their HPV vaccination practices. In implementation science, readiness is conceptualized as organizational commitment to implementing an innovation, including commitment and involvement of managers and leaders (14). In states A and B, where adoption rates were relatively high, coaches presented the visit as a mandate, and in state A, they additionally scheduled visits with medical assistants who had limited authority. Although clinics in these states adopted the HPV vaccination coaching visit, their leadership was not involved in the decision making, and the clinics might not have been ready to implement change. In state C, where the adoption rate was low, QI coaches had to work through multiple levels of network hierarchy to gain access to clinics. The extra effort required to get network leadership to approve HPV vaccination QI coaching might explain the success those coaches had engaging multiple staff to participate in the coaching visit. Coaches in state C chose not to present coaching as a mandate but instead focused on the benefits of the visit to patient care, which possibly contributed to higher levels of commitment to and participation in the coaching visit. Our findings have implications for the design of future QI coaching initiatives. Careful thought needs to be given to how coaches recruit clinics to ensure that clinics are committed to engaging providers and staff in efforts to improve their HPV vaccination processes. The format of the coaching visit might need refinement to take advantage of the finding that providers often join the visit only briefly. For example, the QI coach may include a brief visit with providers as part of a longer visit with staff. Lastly, as clinic networks continue to grow, QI coaches will need to learn how to effectively engage with these networks.

QI coaches in all 3 states demonstrated high fidelity to some HPV vaccination coaching tools and protocols and mixed or low fidelity to others. Coaches’ knowledge of and attitudes toward the tools were central factors influencing fidelity. In all 3 states, QI coaches reported consistent use of the report card and goal setting, which they perceived to be clear and concise. Coaches also valued the content of the presentation slides, particularly the concise presentation of evidence supporting HPV vaccination. Fidelity using the presentation slides was mixed, however, with coaches in 1 state converting slides into a 1-page hand-out. In the other 2 states, coaches reported skipping information or sections of the presentation that were not applicable to staff participating in the coaching visit.

Coaches reported limited fidelity to HPV vaccination coaching protocols for selecting QI strategies and action planning. Although coaches engaged clinic staff in strategy selection, they often promoted the strategies recommended by the traditional AFIX program, rather than the shorter list of strategies included in the HPV vaccination coaching protocols. This highlights the importance of providing booster trainings to ensure that all QI coaches are knowledgeable about the tools and protocols. QI coaches reported that they did little action planning, largely because the staff person participating in the coaching visit lacked the authority to do so. The lack of fidelity to action planning provides further support for the importance of recruiting clinics that are ready to improve HPV vaccination coverage, and, therefore, have the capacity and motivation to develop an action plan. Lastly, study findings on fidelity raise the question of when adaptation might be appropriate to improve the suitability of a tool or protocol to the needs of a particular state or clinic, and when adaptation is not appropriate because it alters 1 of the intervention’s 4 core components. Further research is needed to answer questions about which aspects of HPV vaccination QI coaching are essential to its effectiveness and which can be adapted.

Our study had several limitations. The HPV vaccination QI coaching intervention was evaluated in only 3 states; therefore, the ability to generalize the findings to other states remains to be established. Factors identified to explain variations in implementation outcomes are based on QI coaches’ perceptions; therefore, they are exploratory. Further study is needed to establish which factors influence QI implementation outcomes as well as the impact of those outcomes on vaccine coverage rates.

One of our intervention’s strengths was its alignment of tools and protocols with widely recognized QI coaching competencies, including skill in using data to drive change, knowledge of the evidence base informing change, familiarity with strategies to implement change, and skills in communication and relationship building. These 4 competencies provide a foundation for exploring core components of QI coaching. We found that QI coaches maintained fidelity to tools and protocols related to using data to drive change (report card, goal setting). They also maintained fidelity to the presentation of the evidence supporting the change. Even when they switched content to a 1-page format, they retained focus on informing clinics of the evidence base supporting HPV vaccination. Fidelity results were mixed for tools and protocols related to strategies to implement change and begin action planning. This is a concern, because evidence suggests that using data to drive change is most effective when it is coupled with guidance on QI and other change strategies (10). Findings also point to the importance of designing tools and protocols to support communication and relationship building. Careful consideration needs to be given to ensuring that QI coaches engage someone in the clinic (or delivery system hierarchy) who will verify the clinic’s readiness to improve HPV vaccination rates and engage providers and staff in the HPV vaccination QI coaching visit.
